# Metacognition and Self-Disturbances in Schizophrenia: Implications for Psychological and Neurocognitive Therapy

**DOI:** 10.3390/brainsci16090956

**Published:** 2026-09-09

**Authors:** Natasza Orlov, Izabela Sarzyńska, Jolanta Góral-Półrola, Patrycja Leśnicka, Oliwia Bartkowska, Szymon Stańczyk, Ewa Siwiec, Artur Ziółkowski, Marta Kopańska

**Affiliations:** 1 Department of Medical Communication and Professional Competency Development, Faculty of Medicine, University of Rzeszów, 35-959 Rzeszów, Poland; norlov@ur.edu.pl (N.O.); patrycjazagata@gmail.com (P.L.); 2 Student Research Club “Reh-Tech”, Faculty of Medicine, University of Rzeszów, 35-959 Rzeszów, Poland; sarzynskaizabela-2001@wp.pl (I.S.); ob130723@stud.ur.edu.pl (O.B.); ss136030@stud.ur.edu.pl (S.S.); 3 Faculty of Pedagogy and Psychology, Jan Kochanowski University of Kielce, 25-029 Kielce, Poland; jolagoralpolrola@gmail.com; 4 Department of Psychology, University College of Professional Education in Wroclaw, 53-329 Wrocław, Poland; ewa.siwiec@wskz.pl (E.S.); artur.ziolkowski@wskz.pl (A.Z.)

**Keywords:** schizophrenia, metacognition, self-disturbance, minimal self, MERIT, Metacognitive Training, cognitive remediation, recovery

## Abstract

**Highlights:**

**What are the main findings?**

- Self-disturbances and metacognitive deficits are related dimensions that may contribute to the clinical presentation and functional outcomes of schizophrenia.
- Integrating knowledge on self-disturbances with contemporary models of metacognition provides a better understanding of the mechanisms underlying schizophrenia and its clinical presentation.

**What are the implications of the main findings?**

- Assessment of self-disturbances and metacognitive functioning may complement standard clinical evaluation and support more individualized treatment planning; however, its additional diagnostic value requires further investigation.
- Interventions targeting metacognition, such as Metacognitive Reflection and Insight Therapy (MERIT), Metacognitive Training (MCT), and Cognitive Remediation, may improve psychosocial functioning and support recovery.

**Abstract:**

**Background/Objectives**: Schizophrenia is a complex psychiatric disorder characterized by positive, negative, and cognitive symptoms, as well as disturbances of the self and metacognitive dysfunction. Increasing evidence suggests that these dimensions contribute substantially to psychosocial functioning and recovery, yet they are often investigated separately. This narrative review aims to integrate current knowledge on self-disturbances and metacognition in schizophrenia and discuss their relevance for contemporary therapeutic approaches. This review proposes an integrative conceptual framework positioning disturbances of the minimal self and metacognitive dysfunction as complementary dimensions that may contribute to schizophrenia psychopathology and may have relevance for recovery-oriented interventions. **Methods**: A narrative review of the peer-reviewed literature was conducted. Classical theoretical papers, recent empirical studies, systematic reviews, and meta-analyses addressing self-disturbances, metacognition, and therapeutic interventions in schizophrenia spectrum disorders were analyzed. **Results**: Current evidence indicates that disturbances of the minimal self and metacognitive dysfunction may represent complementary and interacting dimensions of schizophrenia psychopathology. Self-disturbances affect the subjective sense of identity, agency, and self-presence, whereas metacognitive deficits impair the integration of information about oneself and others into coherent mental representations. These impairments are associated with poorer psychosocial functioning, reduced quality of life, and limited recovery. Interventions targeting metacognitive functioning, including Metacognitive Reflection and Insight Therapy, Metacognitive Training, and cognitive remediation, have shown promising effects on functional outcomes and recovery. **Conclusions**: Integrating phenomenological concepts of self-disturbance with contemporary metacognitive models may provide a broader understanding of schizophrenia than symptom-based approaches alone. In clinical practice, instruments such as the Examination of Anomalous Self-Experience (EASE) and Metacognition Assessment Scale–Abbreviated (MAS-A) may complement standard assessment by providing information about anomalous self-experiences, insight, and psychosocial functioning. However, the additional diagnostic value of combining these assessments with established clinical procedures has not yet been clearly demonstrated. They should therefore be regarded as supplementary tools that may inform clinical formulation and individualized, recovery-oriented treatment planning rather than as standalone diagnostic measures.

## 1. Introduction

Schizophrenia is a chronic, heterogeneous mental disorder that remains one of the greatest challenges in contemporary psychiatry. The disorder affects cognitive processes, perception, emotional regulation, and behavior, leading to significant impairment in psychosocial functioning. Schizophrenia has a global lifetime prevalence of approximately 0.6%, although prevalence varies across populations and geographical regions [1]. It most commonly manifests during late adolescence or early adulthood, when the first psychotic symptoms appear [2]. Although the risk of developing schizophrenia is very similar in both sexes, the disorder is more common in men before the age of 40 [3]. The disorder is associated with increased mortality and an elevated risk of suicide [4,5]. Individuals with schizophrenia have an approximately 2–3-fold higher risk of premature death compared with the general population, and their average life expectancy is significantly reduced compared with the general population [6,7]. The etiology of schizophrenia is complex. Among the factors responsible for the development of schizophrenia, genetic predisposition plays the best-documented role. The disorder has a complex, polygenic basis and is characterized by high heritability, estimated at 60–80% [8,9]. Environmental factors that adversely affect the brain and nervous system also contribute to the development of this disorder. These factors may include complications during pregnancy and childbirth, advanced parental age, particularly paternal age, infections, adverse childhood experiences, and the use of psychoactive substances [10,11]. These factors are associated with abnormalities in the functioning of brain networks, as well as alterations in synaptic plasticity and myelination [12]. Consequently, schizophrenia leads to persistent impairments in functioning across multiple areas of life, including social relationships, education, occupational activity, and independent living. The disorder constitutes a significant burden not only for individuals with schizophrenia and their families but also for healthcare and social care systems. Despite advances in diagnostics, pharmacotherapy, and psychosocial interventions, many individuals with schizophrenia still do not achieve satisfactory functional improvement or sustained recovery, highlighting the need for a better understanding of the mechanisms underlying schizophrenia [13,14,15].

Schizophrenia is characterized by the presence of positive and negative symptoms, as well as cognitive impairments. Positive symptoms include delusions, hallucinations, behavioral disturbances, and speech disturbances. Negative symptoms include avolition, alogia, anhedonia, aversion, and social withdrawal [16,17].

Classical models of schizophrenia, based primarily on the presence of clinical symptoms, neurobiology, or cognitive deficits, do not fully capture the subjective experience of schizophrenia. Increasing attention is currently being paid to metacognition as well as disturbances of the sense of self [18,19,20]. Metacognition is a cognitive process that supports continuous self-reflection, adaptation, and collaboration with others throughout life. It enables individuals to experience themselves and others and to interpret prereflective, reflective, embodied, and cognitive experiences. It is also defined as the human capacity to monitor one’s own mental states and processes, or as “thinking about thinking” [21,22,23]. Research shows that individuals with schizophrenia experience metacognitive deficits, including difficulties in understanding and integrating experiences related to themselves and others, as well as in using this knowledge to cope adaptively with everyday life challenges [24]. At the same time, individuals with schizophrenia exhibit cognitive impairments, including deficits in reasoning, abstract thinking, planning, and problem-solving, working memory, visual and verbal learning, as well as impairments in social cognition. Furthermore, they may experience difficulties in developing a coherent understanding of their own emotions and beliefs, as well as in understanding the emotions, perspectives, and needs of others [25,26,27,28]. An important dimension of schizophrenia is the disturbance of the sense of self. The concept of the self refers to the conscious experience of one’s life from a first-person perspective, as a self-present, temporally enduring, singular, and embodied subject of experience and action [29,30]. Schizophrenia is associated with disturbances of the sense of self. In other words, individuals with schizophrenia may experience a disruption of the natural and automatic sense of being the author of their own thoughts and actions. For example, thoughts may no longer be perceived as internally generated, which may contribute to experiences in which thoughts are perceived as being inserted from an external source. Individuals with schizophrenia may also experience difficulties engaging in life, leading to a sense of being absent from themselves, as well as a blurring of the boundaries between the self and the external world [30,31,32]. Currently, there is growing interest in the use of modern therapeutic approaches for individuals diagnosed with schizophrenia, such as Metacognitive Reflection and Insight Therapy (MERIT) and Metacognitive Training (MCT), which aim to improve metacognitive abilities, enhance the perception of oneself and others, and increase awareness of cognitive biases and the fallibility of cognitive processes [33,34].

Although disturbances of the sense of self and metacognitive deficits have increasingly become the focus of schizophrenia research, most existing studies examine these phenomena separately. There remains a lack of reviews integrating current knowledge on disturbances of the sense of self, metacognition, and cognitive functioning, as well as their significance for the diagnosis and treatment of schizophrenia.

The aim of this review is to analyze metacognitive mechanisms and disturbances of the sense of self in individuals with schizophrenia. It examines how individuals with schizophrenia perceive and interpret their own mental states, including difficulties in recognizing thoughts as internally generated products of the mind and in distinguishing their own perspective from that of others. Particular attention is given to disturbances of ipseity, manifested by a blurring of the boundaries between the inner self and the external environment. An important objective of this review is also to present the therapeutic implications of these findings. Contemporary approaches, including Metacognitive Reflection and Insight Therapy (MERIT), Metacognitive Training (MCT), and neurocognitive rehabilitation, are aimed at improving cognitive processes, supporting the subjective sense of identity, and enhancing the everyday functioning of individuals with schizophrenia.

## 2. Materials and Methods

### 2.1. Review Methodology

Given the complex and multidimensional nature of the topic, encompassing the psychopathology of schizophrenia, phenomenological approaches to self-disturbance, metacognition, and therapeutic interventions, a narrative review was conducted. This approach enabled the integration of findings from different theoretical and methodological perspectives and examination of the relationships between disturbances of the sense of self, metacognitive deficits, and their clinical and therapeutic significance.

### 2.2. Literature Search

Studies in the literature relevant to the scope of the review were identified through searches of PubMed, Scopus, and Web of Science. No publication date restrictions were applied, allowing both classical phenomenological contributions and recent empirical studies to be considered. The review focused on publications written in English. Searches used combinations of terms covering three main areas:

(1) Population: Schizophrenia and schizophrenia spectrum disorders;
(2) Phenomena of interest: Self-disorder, self-disturbance, minimal self, ipseity, metacognition, and metacognitive deficits;
(3) Therapeutic interventions and treatment outcomes: Metacognitive Reflection and Insight Therapy (MERIT), Metacognitive Training (MCT), cognitive remediation, and recovery.

Additional relevant studies were identified by examining the reference lists of selected review articles, meta-analyses, and key papers addressing the topics covered in this review.

As this was a narrative review, no formal assessment of the methodological quality or risk of bias of the selected publications was conducted.

## 3. Disturbances of the Sense of Self in Schizophrenia

### 3.1. The Concept of the Self and Its Significance in Schizophrenia

The concept of disturbances of the sense of self (the sense of “I”) in schizophrenia originates from the classical works of Emil Kraepelin and Eugen Bleuler, who, as early as the beginning of the twentieth century, identified disturbances in the unity of personality and the integrity of experience as fundamental features of the clinical presentation of the disorder [35]. Currently, researchers support these observations, indicating that these disturbances are not limited to changes in the content of thought or perception but instead involve a more fundamental level of mental functioning. In other words, they affect the way individuals with schizophrenia experience themselves [36]. The sense of self refers to the psychological processes through which individuals perceive themselves as distinct, coherent, and unique persons, maintaining a continuous sense of personal identity over time [29]. One of the most widely recognized models of the sense of self is the concept proposed by Blanke, according to which it comprises four fundamental components: the sense of body ownership, awareness of the body’s location in space, the experience of reality from a first-person perspective, and the belief that one is in control of one’s own actions, referred to as a sense of agency [37]. A similar account is offered by Jimenez and Green, who distinguish between two aspects of the self. The first, referred to as the structural self, includes the sense of body ownership and agency. The second, termed the experiential self, involves self-reflection, autobiographical memory, and the ability to identify the source of information. According to the authors, both aspects are disrupted in schizophrenia, which may make it difficult to distinguish one’s own thoughts and experiences from information originating in the external world [38].

Contemporary models conceptualize the sense of self as a multidimensional construct. In schizophrenia, particular importance is attributed to two levels: the minimal self (minimal self or ipseity) and the narrative self. The most fundamental level is the minimal self (minimal self or ipseity). It can be understood as the prereflective, automatic sense of being the subject of one’s own thoughts, emotions, and actions. Under normal conditions, it provides continuity of experience and the natural conviction that one’s experiences belong to the same person. The most complex level is the narrative self, which encompasses an individual’s life history, personality traits, beliefs, social roles, and personal identity [39]. In turn, Yeh et al. emphasized that the minimal self is closely linked to episodic memory, which enables individuals to recall their own past experiences [29].

An increasing body of evidence indicates that disturbances of the minimal self are among the most characteristic features of schizophrenia and may emerge at very early stages of psychosis, preceding the onset of full-blown psychotic symptoms [40]. For this reason, disturbances of the sense of self are considered one of the key features of the schizophrenia spectrum phenotype [41].

The clinical significance of the concept of self-disturbances is supported by studies using the Examination of Anomalous Self-Experience (EASE) scale, which is currently one of the most widely used instruments for assessing anomalous self-experiences in individuals with schizophrenia spectrum disorders (SSDs) [30]. In a study involving 226 individuals with various psychiatric disorders, Nordgaard et al. demonstrated that the EASE scale showed high sensitivity and specificity in identifying SSDs [42]. These findings are consistent with meta-analytic evidence indicating that self-disturbances occur at substantially higher levels in SSDs. However, anomalous self-experiences are not exclusive to SSDs and have been reported in other psychiatric populations. In a study involving 54 participants, disturbances related to the boundary between the self and the external world were found to be more pronounced in individuals with substance-induced psychosis, whereas disturbances involving self-awareness and the sense of presence were more frequently observed in individuals with SSDs [43]. It is worth noting that the assessment of disturbances of the sense of self may provide a valuable complement to contemporary schizophrenia diagnostics and may facilitate the identification of individuals at increased risk of developing psychosis.

### 3.2. Clinical Presentation of Self-Disturbances in Schizophrenia

Disturbances of the sense of self manifest through a range of characteristic experiences. Individuals with schizophrenia frequently describe a diminished sense of personal identity, feelings of alienation, and a perception of being fundamentally different from other people. They often experience a loss of their natural sense of being present in the world, disturbances in the continuity of their thoughts and experiences, as well as difficulties in maintaining a coherent sense of self [31]. Unlike delusions and hallucinations, which emerge during the acute phase of illness, self-disturbances may be observed prior to illness onset. In a study involving 87 non-psychotic adolescents, disturbances of the basic sense of self were associated with prodromal symptoms and greater clinical impairment. Longitudinal evidence further indicates that basic self-disturbances in adolescence may predict the later development of SSDs [44]. These findings suggest that their assessment may contribute to the identification of individuals at risk of developing schizophrenia [45].

One of the most characteristic manifestations of self-disturbances is hyperreflexivity, defined as an excessive awareness of mental processes that, in healthy individuals, occur automatically and remain outside conscious control. Fryaerts defines hyperreflexivity as an excessive focus on one’s own thoughts, sensations, and actions. Processes that normally occur automatically become the subject of conscious attention and analysis. Individuals with SSDs report that they begin to observe their own thoughts excessively or consciously monitor activities that they had previously performed without deliberate reflection [41].

Studies show that individuals with schizophrenia simultaneously exhibit **diminished self-affection**, which leads to a gradual loss of the sense that their own thoughts, emotions, and actions genuinely belong to them. Patients frequently describe feelings of alienation, a diminished sense of presence in their own lives, and difficulties recognizing their thoughts as originating from their own minds. These changes may precede the development of more pronounced psychotic symptoms. **Diminished self-affection** may also affect everyday functioning by impairing the ability to establish interpersonal relationships, make decisions, and maintain a coherent sense of self, thereby contributing to social withdrawal and a reduced quality of life [31].

As the disorder progresses, the boundary between the inner world and the external environment gradually becomes blurred. These disturbances may lead to difficulties in distinguishing one’s own mental processes from external stimuli, resulting in the development of symptoms such as thought echo, delusions of control, delusions of influence, and auditory hallucinations [46]. The clinical presentation of self-disturbances also includes difficulties in developing a coherent identity, maintaining a continuous sense of self, and establishing interpersonal relationships. Individuals with SSDs frequently describe feelings of alienation, reduced engagement in everyday life, and difficulties interpreting their own experiences and emotions. These disturbances may substantially impair social functioning and quality of life, even in individuals with relatively mild classical psychotic symptoms [47].

The disturbances described above lead to profound changes in the way individuals experience themselves and the surrounding world, constituting one of the most characteristic features of the clinical presentation of schizophrenia.

Disturbances of the boundary between the self and the external world are particularly evident in Schneiderian first-rank symptoms, such as thought insertion, thought broadcasting, and delusions of control. In their article, Jimenez and Green describe two contrasting patterns of this disturbance. A diminished sense of self may make it difficult to distinguish one’s own experiences from external influences. As a result, individuals with SSDs may perceive their thoughts or actions as alien or imposed by someone else. In contrast, an exaggerated sense of self may lead external events to be interpreted as directly related to oneself or as threatening [38].

### 3.3. Clinical Significance of Self-Disturbances in Schizophrenia

An increasing body of evidence indicates that disturbances of the sense of self are important not only for understanding the psychopathology of schizophrenia but also for contemporary clinical practice. One of the most important clinical applications of self-disturbances is their potential to facilitate the early detection of schizophrenia. A growing number of studies suggest that anomalous self-experiences emerge before the onset of full-blown psychosis. Consequently, their assessment may support the implementation of appropriate therapeutic interventions before the first psychotic episode develops. In addition, self-disturbances may also have prognostic value [31]. Studies indicate that self-disturbances persist over extended periods and exhibit relatively high stability, similar to the negative symptoms of schizophrenia. Their presence and severity may provide valuable information regarding the future course of the disorder, the risk of relapse, and the extent of deterioration in psychosocial functioning [47,48]. Raballo et al. observed that the assessment of self-disturbances may contribute to diagnostic characterization/differentiation. Considering anomalous self-experiences provides a better understanding of the mechanisms underlying schizophrenia and facilitates its differentiation from other psychiatric disorders [31]. However, although self-disturbances have been associated with subsequent development of SSDs in clinical high-risk and help-seeking populations, evidence that their assessment provides incremental predictive value beyond established clinical risk measures remains limited. Their potential clinical utility should therefore be considered complementary to, rather than as a replacement for, established risk-assessment approaches.

Growing interest in self-disturbances also stems from their potential significance for research into the etiology of schizophrenia. They are increasingly regarded as an important phenotypic marker that may support investigations into the genetic and neurodevelopmental mechanisms underlying the disorder. A better understanding of self-disturbances may contribute to the development of more accurate models for predicting the risk of schizophrenia and to the advancement of more personalized therapeutic approaches [40,45].

The main dimensions of self-disturbances in schizophrenia, together with their principal characteristics, clinical manifestations, and clinical significance, are summarized in Table 1.

## 4. Metacognition in Schizophrenia

### 4.1. Definition and Models of Metacognition

Metacognition is a complex psychological construct that refers to the ability to monitor, interpret, and regulate one’s own cognitive processes. In Flavell’s classical conceptualization, the term primarily referred to “thinking about thinking”. However, contemporary models have substantially expanded its scope. Metacognition is now understood as the ability to integrate information about one’s own thoughts, emotions, and experiences with knowledge of the mental states of others, thereby enabling the development of a coherent understanding of oneself and the surrounding world [49,50,51].

Whereas the traditional assessment of cognitive deficits (as reflected, for example, in the Measurement and Treatment Research to Improve Cognition in Schizophrenia (MATRICS) initiative) focuses on domains such as processing speed, attention, and working memory, findings from recent studies indicate that metacognition represents a distinct cognitive construct [52,53,54]. Studies based on the conceptual framework proposed by Semerari et al. describe metacognition as the ability to understand and integrate one’s own mental states as well as those of others, extending beyond basic neurocognitive functions [50].

To facilitate the distinction between these related but conceptually different domains, the main characteristics of neurocognition, social cognition, and metacognition are summarized in Table 2.

In recent years, increasing emphasis has been placed on the fact that metacognition is not simply the sum of individual cognitive functions. Even individuals who perform relatively well on neuropsychological tests may experience considerable difficulties in constructing a coherent narrative of their own lives, understanding the motives underlying their behavior, or interpreting the intentions of others [56]. This indicates that intact memory, attention, or executive functioning does not necessarily guarantee preserved metacognitive abilities, which represent a distinct dimension of psychological functioning [56].

One of the best-described models is the concept proposed by Lysaker and colleagues, according to which metacognition has a hierarchical structure. It encompasses the ability to recognize individual mental states, integrate them into more complex representations of oneself and others, and use this knowledge to make adaptive decisions. This model places particular emphasis on the capacity to develop a coherent sense of identity and to respond flexibly to changing social situations, both of which are considered essential for the recovery process in individuals with schizophrenia.

It is also important to distinguish metacognition from social cognition. Social cognition encompasses processes such as emotion recognition, theory of mind, and the perception of social cues, whereas metacognition integrates this information with autobiographical and emotional experiences, enabling its practical application. Thus, metacognitive functioning involves not only recognizing the emotions of others but also understanding their significance within the context of one’s own experiences and interpersonal relationships [57].

A growing body of evidence indicates that the level of metacognitive functioning is associated with the severity of negative symptoms, social functioning, quality of life, and the effectiveness of psychotherapeutic interventions. For this reason, metacognition is currently regarded as one of the most important mechanisms influencing the course of schizophrenia and has become a major target of contemporary therapeutic interventions aimed at promoting recovery [58].

### 4.2. Metacognitive Deficits in Schizophrenia

Individuals with SSDs exhibit deficits across four fundamental domains of metacognition: self-reflectivity, decentration (the ability to adopt the perspectives of others), understanding the mental states of others, and mastery, defined as the ability to use knowledge of one’s own experiences to cope with everyday challenges and solve problems [50]. Within this framework, decentration refers specifically to the capacity to recognize that others have perspectives, motives, and mental states that are independent of oneself. Although this overlaps with perspective-taking and theory of mind in social cognition, it differs in emphasizing the integration of this understanding within a broader representation of interpersonal experience rather than the identification or inference of another person’s mental state alone. These deficits persist regardless of the severity of clinical symptoms and are more pronounced than those observed both in healthy individuals and in individuals with other serious mental illnesses [59].

One of the most widely used instruments for assessing metacognitive functioning in individuals with schizophrenia is the Metacognition Assessment Scale–Abbreviated (MAS-A), developed by Lysaker and colleagues based on the earlier conceptual framework proposed by Semerari and colleagues. The scale enables a qualitative assessment of the level of integration of psychological experiences through the analysis of narratives, encompassing four core domains of metacognition: self-reflectivity, understanding the mind of others, decentration, and mastery, defined as the ability to use knowledge of one’s own experiences to effectively cope with everyday problems and challenges [50,60]. Unlike conventional neuropsychological tests, the MAS-A does not assess the speed or accuracy of cognitive task performance but rather the extent to which individuals are able to construct coherent and integrated representations of themselves and others. Consequently, this instrument can capture disturbances in psychological functioning that often remain undetected during standard neurocognitive assessment [56]. Studies using the MAS-A indicate that individuals with schizophrenia show impairments across all four domains, with particularly marked difficulties in developing a coherent representation of the self and using this knowledge to manage everyday problems [51,56].

Studies using the MAS-A have consistently demonstrated that lower levels of metacognitive functioning are associated with greater severity of negative symptoms, poorer social functioning, reduced insight into illness, and less favorable outcomes of psychiatric rehabilitation [51,61,62]. An increasing number of authors have also emphasized the high clinical utility of the MAS-A in monitoring the effects of psychotherapies aimed at enhancing metacognitive abilities, particularly Metacognitive Reflection and Insight Therapy (MERIT), in which improvements in MAS-A scores are considered one of the primary indicators of treatment effectiveness [51].

Deficits in self-reflectivity make it difficult for individuals to recognize and interpret their own emotions, beliefs, and motivations. As a result, internal experiences become fragmented and difficult to organize, which may contribute to distorted interpretations of reality and the persistence of psychotic symptoms. At the same time, a reduced capacity to adopt the perspectives of others leads to difficulties in understanding their intentions and social behavior, increasing the risk of misunderstandings, social withdrawal, and interpersonal conflicts.

Particular clinical importance is attributed to impairments in the domain of mastery, which enables individuals to use knowledge about themselves to respond effectively to stressful situations. Many individuals retain the ability to recognize their own experiences while being unable to use this knowledge to regulate emotions or plan their behavior. These limitations are associated with reduced independence, difficulties adapting to everyday challenges, and poorer outcomes of psychosocial rehabilitation.

A growing body of evidence also indicates that metacognitive deficits are associated with the severity of both positive and negative symptoms of schizophrenia. Impaired integration of experiences may contribute to the development of delusions and hallucinations by hindering the ability to distinguish internal experiences from stimuli originating in the external environment. Likewise, a diminished capacity to construct a coherent representation of the self has been linked to greater apathy, anhedonia, and social withdrawal, which are characteristic negative symptoms. Recent meta-analyses further suggest that the level of metacognitive functioning may be an important predictor of long-term psychosocial functioning and the effectiveness of therapeutic interventions.

It is important to emphasize that metacognitive deficits are not merely a consequence of the chronic course of the illness. Increasing evidence indicates that they are already present during the early stages of schizophrenia and even among individuals at clinical high risk for psychosis. These findings suggest that metacognitive disturbances may represent one of the core psychopathological mechanisms underlying schizophrenia and that their identification and treatment should be incorporated into early psychiatric intervention.

### 4.3. Impact of Metacognitive Deficits on Functioning

Metacognitive deficits are among the most important factors determining the functioning of individuals with schizophrenia and are currently recognized as one of the key mechanisms influencing the course of the disorder and the recovery process [51,56]. Unlike classical neurocognitive deficits, which primarily affect processing speed, working memory, and executive functioning, metacognitive deficits impair the ability to integrate cognitive, emotional, and interpersonal experiences into a coherent representation of oneself and the surrounding world [50,62]. A growing body of evidence indicates that the level of metacognitive functioning is an important predictor of everyday functioning beyond performance on traditional neuropsychological tests [51,56].

The most evident consequences of metacognitive deficits are observed in psychosocial functioning. A reduced capacity to understand one’s own mental states and to adopt the perspectives of others impairs the ability to establish and maintain satisfying interpersonal relationships, increases vulnerability to social conflicts, and contributes to social withdrawal [55,56,61]. Individuals with more severe metacognitive impairments also experience greater difficulties in obtaining and maintaining employment, functioning independently, and fulfilling family roles, regardless of the severity of their psychotic symptoms [62]. Findings from longitudinal studies suggest that the level of metacognitive functioning is one of the strongest predictors of long-term psychosocial functioning and quality of life in individuals with schizophrenia.

Increasing attention has also been paid to the relationship between metacognitive deficits and the severity of clinical symptoms. Impaired integration of one’s own experiences may hinder the ability to distinguish internal experiences from stimuli originating in the external environment, thereby contributing to the development and persistence of delusions and auditory hallucinations [51,63]. At the same time, a reduced capacity to construct a coherent representation of the self has been associated with greater severity of negative symptoms, including avolition, anhedonia, and social withdrawal, which are the primary contributors to long-term functional impairment in individuals with schizophrenia [56]. Contemporary meta-analyses indicate that metacognition partially mediates the relationship between neurocognition, social cognition, and social functioning, representing an important mechanism underlying the heterogeneity of the clinical presentation of schizophrenia [51].

An important clinical aspect is the influence of metacognition on insight into illness and treatment adherence. Individuals with more severe metacognitive deficits are more likely to have difficulty recognizing their symptoms as manifestations of a mental disorder, which reduces motivation for treatment and increases the risk of poor medication adherence and psychotic relapse [61]. These disturbances also affect the way individuals interpret feedback received from others, making it more difficult to modify maladaptive beliefs and cognitive schemas [50,63]. As a result, metacognitive deficits may contribute to the persistence of distorted interpretations of reality and the exacerbation of difficulties in adaptation.

In recent years, increasing attention has been paid to the role of metacognition in the recovery process, understood as achieving a satisfying and autonomous life despite the persistence of some symptoms of the disorder. Higher levels of metacognitive functioning have been associated with a greater sense of agency, better emotion regulation, more realistic goal setting, and greater engagement in the therapeutic process [51,56]. Individuals who are able to construct a more integrated narrative of their own lives demonstrate greater psychological resilience and cope more effectively with the challenges of everyday functioning, which is associated with a higher quality of life and a more favorable prognosis [56].

A growing body of evidence also indicates that metacognition represents a promising and modifiable therapeutic target. Interventions aimed at enhancing self-reflectivity, understanding the perspectives of others, and integrating psychological experiences, such as Metacognitive Reflection and Insight Therapy (MERIT) and Metacognitive Training (MCT), have been shown to improve psychosocial functioning, enhance the sense of agency, and reduce the severity of psychotic symptoms [51]. Findings from recent meta-analyses suggest that improvements in metacognitive abilities may represent one of the mechanisms responsible for the sustained effects of psychotherapy. These findings underscore the importance of incorporating metacognitive assessment into both the diagnostic process and the planning of comprehensive treatment for individuals with schizophrenia [64].

## 5. Relationship Between Self-Disturbances and Metacognition

The evidence reviewed suggests that metacognitive deficits and self-disturbances represent clinically relevant and potentially interacting dimensions of schizophrenia psychopathology. The extent to which these phenomena exert reciprocal influences, however, remains uncertain, as the currently available evidence is largely cross-sectional and correlational, making it difficult to establish causality and temporal sequence [19].

Nevertheless, a potentially bidirectional relationship provides a useful framework for considering how disturbances at different levels of self-experience may interact. Disturbances of the minimal self may weaken the fundamental sense of being the subject of one’s own experiences, whereas impaired metacognitive abilities could hinder the integration of these experiences into a coherent representation of oneself and the surrounding world [56,65]. The phenomenological model of schizophrenia, developed by Sass and Parnas, proposes that the primary disturbance is the loss of the prereflective sense of self (the minimal self), referred to as a disturbance of ipseity. As a result, mental processes that normally occur automatically become the object of conscious monitoring and excessive self-observation, giving rise to hyperreflexivity. At the same time, there is a diminished sense of self-presence (diminished self-affection), predisposing individuals to experience their own thoughts and perceptions as alien or externally imposed [19,66]. Within this framework, metacognitive impairments are not viewed as independent deficits but rather as developing on the basis of earlier disturbances in the fundamental experience of self.

Conversely, limited metacognitive abilities may further exacerbate self-disturbances. Difficulties in recognizing, organizing, and integrating one’s own experiences may make it difficult for individuals to construct a coherent autobiographical narrative or assign meaning to their lived experiences. Consequently, experiences may remain fragmented and incoherent, which may intensify the sense of psychological disorganization and contribute to the persistence of psychotic symptoms [51,61]. This proposed relationship points to a potential vicious cycle in which self-disturbances may aggravate metacognitive deficits, while impaired metacognition could further destabilize the sense of personal identity.

Neuroimaging findings indicate considerable overlap between the neural systems supporting self-related processing and metacognition. Both processes involve cortical midline structures within the default mode network (DMN), particularly the medial prefrontal cortex (mPFC), posterior cingulate cortex (PCC), and precuneus area, regions implicated in self-referential and internally directed processing [67,68]. In schizophrenia, abnormalities within the DMN have been demonstrated across resting-state and task-based neuroimaging studies. Altered DMN homogeneity involving the mPFC and PCC has been observed in first-episode, drug-naïve individuals with schizophrenia, indicating that these abnormalities are present prior to antipsychotic treatment and cannot be attributed solely to medication effects [69]. Task-based studies have also demonstrated altered recruitment of cortical midline regions during self-reflection in schizophrenia [70]. Medial prefrontal and parietal functional connectivity have been associated with metacognitive capacity in psychosis [68]. Taken together, these findings indicate convergence between the neural systems supporting self-related processing and metacognition and suggest that alterations within the DMN may represent a neurobiological component contributing to the co-occurrence of self-disturbances and metacognitive impairment in schizophrenia.

In recent years, increasing attention has been given to the integrative model proposed by Lysaker and colleagues, according to which metacognition serves as a mechanism that enables the reconstruction of a coherent sense of personal identity. Developing the capacity to reflect upon one’s own experiences and assign meaning may support restoration if a sense of agency and autobiographical continuity is preserved, both of which are considered fundamental components of the recovery process [51]. From this perspective, improvements in metacognitive functioning may not only contribute to reductions in psychotic symptoms but, more importantly, support individuals in regaining a more coherent sense of self and functioning more adaptively in interpersonal relationships.

The findings presented in this review indicate that the simultaneous assessment of self-disturbances and metacognitive functioning provides a more comprehensive understanding of schizophrenia psychopathology than the evaluation of either dimension alone. Integrating the phenomenological perspective with metacognitive models offers a more comprehensive explanation for the heterogeneity of the clinical presentation of schizophrenia and provides a conceptual foundation for contemporary psychological interventions aimed not only at symptom reduction but also at restoring a sense of identity, agency, and autonomy [71,72].

A growing body of evidence further suggests that effective therapeutic interventions should simultaneously support the restoration of the sense of self and the development of metacognitive abilities. This assumption underlies contemporary interventions such as Metacognitive Reflection and Insight Therapy (MERIT) and Metacognitive Training (MCT), which are discussed in the following section [34,56].

**Figure 1:**
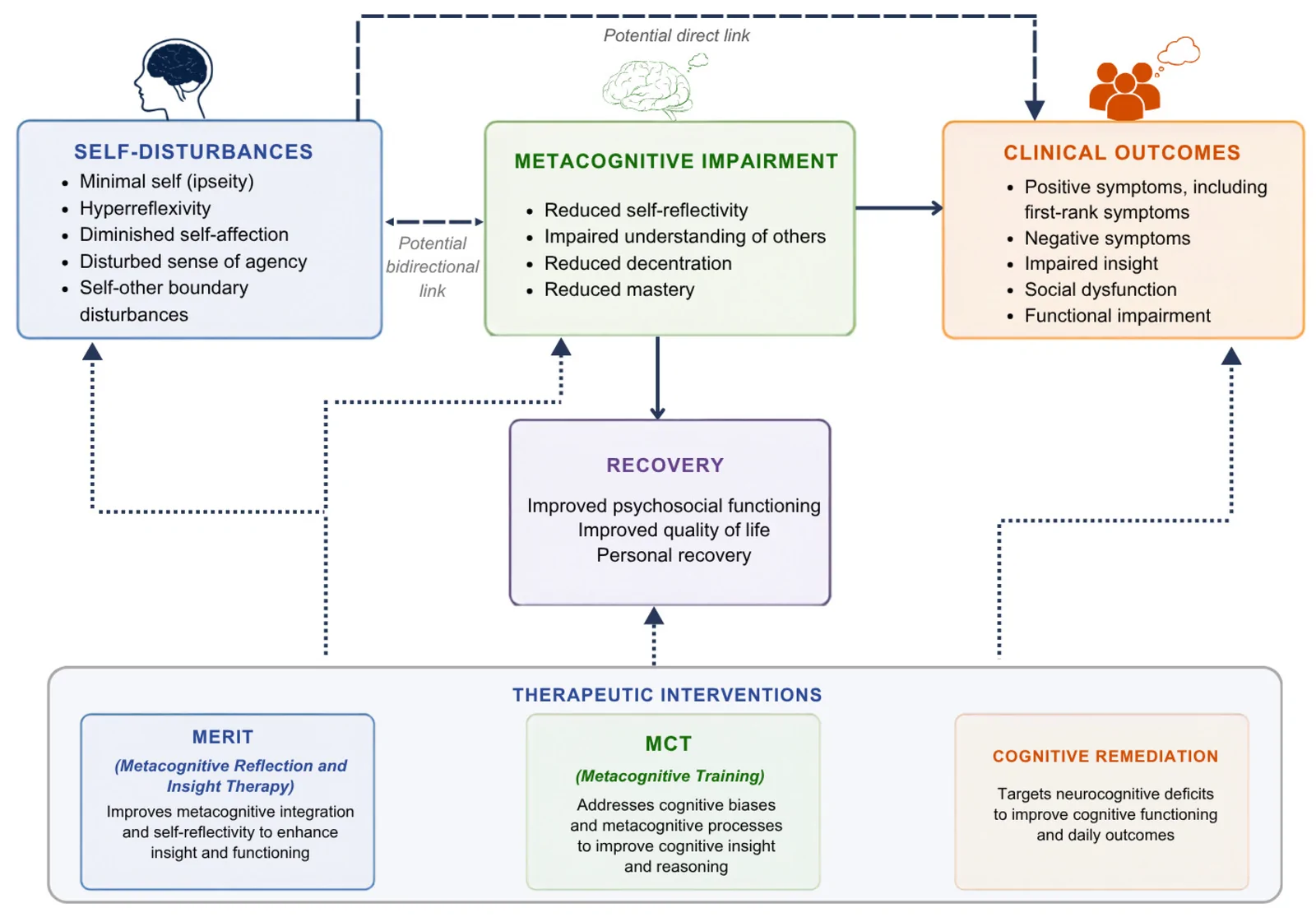
Proposed conceptual model of the relationships between self-disturbances, metacognitive impairment, clinical outcomes, therapeutic interventions, and recovery in schizophrenia. Solid arrows represent the principal relationships considered in the model; dashed arrows indicate potential or hypothetical relationships; and dotted arrows represent therapeutic pathways or targets. The directions of the arrows reflect the proposed model and should not be interpreted as evidence of causality.

The proposed conceptual relationship between self-disturbances, metacognitive impairment, clinical outcomes, recovery, and therapeutic interventions is summarized in Figure 1. The model presented in Figure 1 offers one possible interpretation of the relationships between self-disturbances, metacognition, and symptoms of schizophrenia. Disturbances of the self–other boundary may be directly associated with certain psychotic experiences, including Schneiderian first-rank symptoms [38]. Metacognitive abilities may also influence how individuals understand and organize these experiences. However, it remains unclear whether metacognition actually plays a mediating role or what the direction of these relationships may be. The model should therefore be regarded as a proposal requiring further investigation rather than as an established mechanism of symptom development.

## 6. Therapeutic Implications

Although antipsychotic medication remains the cornerstone of clinical management, many individuals continue to experience impairments in social functioning, occupational performance, interpersonal relationships, and subjective recovery. Current psychological approaches increasingly recognise that effective treatment should extend beyond symptom reduction and address higher-order processes involved in self-reflection, agency, and meaning-making. This perspective has stimulated the development of interventions specifically targeting metacognitive functioning, while also encouraging greater integration between phenomenological concepts of self-disturbance and recovery-oriented models of psychological rehabilitation.

### 6.1. Metacognitive Training (MCT)

Metacognitive Training is the most extensively investigated metacognitive intervention for SSDs. Developed by Moritz and colleagues to target cognitive biases implicated in the development and maintenance of psychotic symptoms, MCT combines psychoeducation with structured cognitive exercises designed to increase awareness of maladaptive thinking patterns and promote more flexible and reflective reasoning [63]. The intervention focuses on modifying the cognitive processes underlying delusions—such as jumping to conclusions, overconfidence in errors, bias against disconfirmatory evidence, and attributional biases—rather than directly challenging the content of beliefs. This process-oriented approach aims to enhance cognitive insight while reducing the resistance that may accompany direct confrontation of beliefs [34,63,65].

To date, more than 40 clinical trials have evaluated the effectiveness of MCT, and at least 10 meta-analyses have examined its clinical efficacy. Overall, the evidence demonstrates high treatment acceptability together with small-to-moderate improvements in positive symptoms, cognitive biases, and cognitive insight, with several studies reporting sustained benefits following treatment [73,74,75,76]. The most recent and comprehensive meta-analyses have largely confirmed these findings, further supporting MCT as an evidence-based adjunctive intervention for SSDs [77].

From a therapeutic perspective, MCT represents an evidence-based intervention that targets the cognitive mechanisms underlying psychotic symptoms rather than their content. By improving awareness of cognitive biases and promoting more flexible reasoning, MCT enhances cognitive insight and facilitates the ability to re-evaluate maladaptive interpretations. Although its effects on symptom severity are generally small to moderate, MCT consistently improves reasoning biases and is well accepted by individuals with SSDs, making it a valuable adjunct to pharmacological treatment and psychosocial rehabilitation [73,74,76,77].

### 6.2. Metacognitive Reflection and Insight Therapy (MERIT)

Metacognitive Reflection and Insight Therapy is a recovery-oriented psychotherapy developed by Lysaker and colleagues that conceptualises metacognition as the capacity to integrate cognitive, emotional, and interpersonal experiences into increasingly coherent and complex representations of oneself and others [61]. MERIT seeks to restore the disrupted meaning-making processes that characterise SSDs by strengthening the individual’s ability to reflect upon, integrate, and use knowledge about their own mental states and those of others. Through collaborative exploration of autobiographical experiences within an intersubjective therapeutic relationship, MERIT aims to enhance self-reflectivity, understanding of others, and the capacity to apply this knowledge to cope with psychosocial challenges and support personal recovery [56,58,61].

A growing body of empirical evidence supports the effectiveness of MERIT in SSDs. Randomised controlled trials have demonstrated improvements in metacognitive capacity, insight, agency, and recognition of cognitive fallibility following treatment [78,79,80]. Additional controlled and open-label studies have reported improvements in subjective recovery, self-reflectivity, psychosocial functioning, and psychotic symptoms, with treatment gains maintained for up to two years following treatment in some cohorts [81,82,83]. Although the current evidence base remains substantially smaller than that supporting MCT, findings consistently indicate that MERIT is a feasible and acceptable intervention that promotes recovery by enhancing metacognitive functioning across diverse clinical presentations.

From a therapeutic perspective, MERIT represents a distinct metacognitive intervention that focuses on rebuilding the capacity to construct integrated representations of oneself and others rather than directly modifying cognitive biases or symptom content. By strengthening metacognitive capacity, the intervention promotes greater self-understanding, personal agency, adaptive problem-solving, and meaningful engagement in recovery. While further large-scale randomised controlled trials are required to establish its comparative effectiveness, current evidence suggests that MERIT offers a promising recovery-oriented approach that complements existing cognitive and psychosocial interventions for SSDs [56,61,79,83].

### 6.3. Neurocognitive Rehabilitation

Cognitive Remediation (CR) is an evidence-based behavioural intervention developed to improve the neurocognitive deficits that are among the strongest predictors of functional disability in SSDs [84]. CR employs structured cognitive exercises targeting domains commonly impaired in schizophrenia, including attention, working memory, executive functioning, and problem-solving, with the aim of improving everyday functioning [85]. While early CR programmes primarily emphasised repeated practice of cognitive tasks, newer approaches increasingly incorporate metacognitive strategy learning, encouraging individuals to monitor their cognitive performance, recognise their cognitive strengths and limitations, develop effective problem-solving strategies, and apply these strategies to real-world situations [84,85,86].

A substantial body of evidence supports the effectiveness of CR in SSDs. To date, the largest meta-analysis, including more than 130 randomised controlled trials and over 8800 participants, demonstrated significant improvements in global cognition and functional outcomes, although improvements in functioning were most consistently observed when CR was integrated with broader psychosocial rehabilitation [87]. These findings are consistent with earlier meta-analyses reporting small-to-moderate improvements in cognitive performance and psychosocial functioning [85,88]. More recent evidence suggests that interventions incorporating therapist-guided cognitive strategy training and techniques promoting the transfer of cognitive skills to everyday life achieve the greatest functional benefits [87,89].

From a therapeutic perspective, CR differs from other metacognitive interventions in that its primary objective is to improve neurocognitive functioning rather than metacognition itself. Nevertheless, metacognitive processes appear to be important for translating cognitive improvements into meaningful functional gains. By promoting self-monitoring, strategic regulation of cognitive performance, and the application of cognitive strategies to everyday challenges, CR facilitates the generalisation of cognitive gains beyond the training environment [84,85,87]. Although the effects on cognition and functioning are generally small to moderate, CR is now considered an evidence-based component of comprehensive rehabilitation programmes for SSDs, particularly when delivered alongside psychosocial interventions that support skill transfer and recovery [85,87].

Taken together, these interventions illustrate the multidimensional nature of metacognition in schizophrenia. MCT primarily targets cognitive biases involved in psychotic reasoning, MERIT seeks to restore higher-order metacognitive capacities underpinning self-reflection and meaning-making, whereas CR incorporates metacognitive strategy learning to promote the transfer of cognitive improvements into everyday functioning. Although differing in their theoretical foundations and therapeutic targets, all three approaches aim to enhance an individual’s capacity to understand, regulate, and adapt to their cognitive and psychological experiences, thereby supporting functional recovery beyond symptom reduction.

Several therapeutic approaches have been developed to address the cognitive, metacognitive, and self-related disturbances observed in schizophrenia. Although these interventions differ in their theoretical foundations and primary therapeutic targets, they share the common goal of improving insight, psychosocial functioning, and recovery. The main characteristics of the principal therapeutic approaches discussed in this review are summarized in Table 3.

## 7. Discussion

### 7.1. The Role of Self-Disturbances and Metacognition in the Psychopathology of Schizophrenia

The findings synthesised in this review support the view that disturbances of the self and metacognitive dysfunction are not independent features of SSDs but interacting dimensions that jointly shape psychopathology, functional disability, and recovery. Rather than representing parallel impairments, disturbances of self-experience and metacognitive dysfunction are hypothesized to reinforce one another, potentially creating a cycle that compromises the integration of subjective experience, social understanding, and adaptive functioning. This integrative perspective extends traditional symptom-based and neurocognitive models by combining phenomenological accounts of subjective experience with cognitive and neurobiological mechanisms, thereby providing a more comprehensive framework for understanding the disorder [41,90].

### 7.2. Clinical and Therapeutic Implications

The findings reviewed in this article suggest that the assessment of disturbances of the self and metacognitive functioning may complement traditional symptom-based approaches to the diagnosis and management of schizophrenia. Although contemporary clinical practice primarily focuses on the severity of positive, negative, and cognitive symptoms, increasing evidence indicates that disturbances of self-experience and metacognitive dysfunction provide additional information regarding illness onset, clinical course, functional outcome, and recovery that is not adequately captured by standard psychopathological or neuropsychological assessments [47].

From a diagnostic perspective, the evaluation of anomalous self-experiences may facilitate the identification of individuals at increased risk of developing SSDs, particularly during the prodromal stages of illness. Instruments such as the EASE have shown potential for differentiating SSDs from other psychiatric conditions, while longitudinal studies indicate that basic self-disturbances may predict the later development of psychosis [35,44,47]. In clinical practice, these domains could be assessed as part of a broader clinical formulation rather than as stand-alone diagnostic markers. The EASE may be used by appropriately trained clinicians to explore anomalous self-experiences through a semi-structured interview, whereas the MAS-A allows metacognitive capacities to be rated on the basis of personal narratives. Information obtained from these assessments may complement standard symptom-based evaluations by providing a more detailed understanding of subjective experience, insight, and psychosocial functioning. However, the extent to which their combined use adds diagnostic value beyond established clinical and risk-assessment procedures has not yet been clearly demonstrated. Their use should therefore be regarded as supplementary rather than diagnostic in itself.

Beyond diagnosis, recognising disturbances of the self and metacognitive dysfunction as clinically relevant therapeutic targets represents a shift from a predominantly symptom-oriented approach towards a recovery-oriented model of care. Recovery in schizophrenia extends beyond a reduction in psychotic symptoms and involves rebuilding a coherent sense of self, strengthening personal agency, improving social functioning, and supporting meaningful participation in everyday life [71,72]. Consequently, comprehensive characterisation of self-experience and metacognitive functioning may help clinicians identify individuals who are most likely to benefit from psychological interventions specifically targeting these domains, thereby improving long-term functional outcomes and quality of life.

The growing evidence reviewed in this article indicates that disturbances of the self and metacognitive dysfunction represent modifiable therapeutic targets rather than fixed features of schizophrenia. While antipsychotic medication remains essential for the management of psychotic symptoms, pharmacological treatment alone is often insufficient to restore social functioning, personal agency, and subjective recovery. Consequently, contemporary treatment models increasingly advocate combining pharmacotherapy with psychological interventions targeting higher-order cognitive and metacognitive processes [34,51,87].

These findings support a shift from a predominantly symptom-centred model of care towards a more holistic and recovery-oriented approach to schizophrenia. Rather than focusing exclusively on reducing psychotic symptoms, treatment should also aim to restore coherent self-experience, strengthen metacognitive capacity, and improve the ability to understand, interpret, and respond adaptively to their own mental experiences and interpersonal environments. Such an approach is consistent with contemporary concepts of recovery, which emphasise autonomy, meaningful participation in everyday life, and long-term functional outcomes alongside clinical remission [71,72].

### 7.3. Future Research Directions and Limitations

Despite the growing body of evidence supporting the role of self-disturbances and metacognitive dysfunction in schizophrenia, several important questions remain unresolved. First, considerable heterogeneity exists in the conceptualisation and assessment of metacognition across studies, making direct comparisons between findings challenging. Although instruments such as MAS-A have become widely used, greater standardisation of assessment methods would facilitate comparisons across studies and strengthen the evidence base [52,59].

Most available studies are cross-sectional and correlational, which prevents firm conclusions about temporal precedence and causality. The proposed bidirectional relationships between self-disturbances and metacognitive dysfunction should therefore be regarded as hypotheses rather than established mechanisms. Longitudinal and intervention studies are needed to determine how these processes may interact during the onset, progression, and recovery stages of schizophrenia [47,48,56].

Another important challenge for future research is the further integration of phenomenological, cognitive, and neurobiological approaches. Although increasing evidence suggests that disturbances of the self and metacognitive dysfunction share common neural mechanisms, particularly involving large-scale brain networks associated with self-referential processing, the precise relationships between subjective experience, brain function, and clinical symptoms remain incompletely understood [91]. Multimodal studies combining neuroimaging, neuropsychological assessment, and detailed evaluation of subjective experience may provide a more comprehensive understanding of these complex interactions.

Finally, further high-quality randomised controlled trials are required to determine the comparative effectiveness of interventions targeting metacognitive functioning and to identify which individuals are most likely to benefit from specific therapeutic approaches. Future research should also explore whether combining pharmacological treatment with interventions such as MCT, MERIT, and CR produces additive or synergistic effects on long-term functional recovery and quality of life [33,85].

The present review has several limitations that should be acknowledged. As a narrative review, it is subject to the inherent limitations of this methodology, including the absence of a formally systematic study selection process, formal risk-of-bias assessment and quantitative synthesis. The literature on self-disturbances is also strongly influenced by particular phenomenological traditions, and the concentration of evidence within these theoretical frameworks should be considered when interpreting the proposed integrative model. Furthermore, the rapidly evolving nature of research on self-disturbances and metacognition means that new evidence may continue to refine current theoretical models and therapeutic recommendations. Nevertheless, by integrating phenomenological, metacognitive, neurocognitive, and therapeutic perspectives, this review provides a comprehensive overview of current knowledge and highlights the importance of considering these complementary dimensions in both research and clinical practice.

## 8. Conclusions

Schizophrenia is increasingly recognized as a disorder extending beyond the traditional framework of positive, negative, and cognitive symptoms. The evidence reviewed in this article indicates that disturbances of the minimal self and metacognitive dysfunction represent complementary dimensions that contribute substantially to the clinical presentation, psychosocial functioning, and recovery of individuals with schizophrenia.

Integrating phenomenological concepts of self-disturbance with contemporary models of metacognition provides a broader understanding of the mechanisms underlying schizophrenia. While self-disturbances reflect fundamental alterations in the pre-reflective experience of the self, metacognitive deficits impair the ability to construct coherent representations of oneself and others. Together, these disturbances influence insight, interpersonal functioning, quality of life, and long-term functional outcomes.

Current evidence also suggests that interventions targeting metacognitive functioning, particularly MERIT, MCT and cognitive remediation incorporating metacognitive strategies, may complement pharmacological treatment by promoting functional recovery rather than symptom reduction alone. A comprehensive therapeutic approach should therefore integrate symptom management with interventions aimed at improving self-experience, metacognitive abilities, and psychosocial functioning.

Although growing evidence supports the clinical relevance of both self-disturbances and metacognition, important gaps remain regarding their neurobiological mechanisms, temporal relationship, and optimal therapeutic targeting. Future longitudinal and intervention studies are needed to clarify these interactions and determine how integrating phenomenological assessment with metacognitive interventions may improve individualized, recovery-oriented care for people with schizophrenia.

## Figures and Tables

**Table 1:** Core dimensions of self-disturbances in schizophrenia and their clinical significance.

Dimension	Characteristics	Clinical Significance	Source
Minimal self (ipseity)	Reduced pre-reflective sense of self and first-person perspective	Core feature of schizophrenia spectrum disorders; may precede psychosis	[36,37,39]
Hyperreflexivity	Excessive awareness of normally implicit mental processes and self-monitoring	Promotes anomalous self-experiences and psychotic symptoms	[41]
Diminished self-affection	Reduced sense of ownership and vitality of subjective experience	Contributes to identity disturbance and impaired psychosocial functioning	[37]
Disturbed sense of agency	Impaired attribution of thoughts and actions to oneself	Associated with passive experiences, positive symptoms, and impaired insight	[37]
Self-world boundary disturbances	Blurred distinction between self and the external world	Contributes to hallucinations, impaired reality testing, and social dysfunction	[43,45,46]

**Table 2:** Comparison of neurocognition, social cognition, and metacognition in schizophrenia.

Domain	Definition	Main Deficits	Clinical Relevance	Source
Neurocognition	Basic cognitive processes such as attention, memory, processing speed, and executive functioning.	Deficits in memory, attention, executive functions, and processing speed.	Associated with functional disability but only partially explains real-world functioning.	[26,27]
Social cognition	Mental processes involved in perceiving and interpreting social information.	Impaired emotion recognition, theory of mind, and social perception.	Predicts social functioning and interpersonal outcomes.	[21,55]
Metacognition	Capacity to understand and integrate one’s own and others’ mental states into a coherent representation.	Impaired self-reflectivity, understanding of others, decentration, and mastery.	Strong predictor of recovery, insight, psychosocial functioning, and treatment outcomes.	[51,56]

**Table 3:** Therapeutic approaches targeting self-disturbances and metacognitive deficits in schizophrenia.

Intervention	Primary Target	Main Outcomes	Source
MERIT	Metacognitive integration and sense of self.	Improved metacognitive capacity, insight, recovery, and psychosocial functioning.	[58,61]
Metacognitive Training (MCT)	Cognitive biases and distorted reasoning.	Reduced positive symptoms, improved cognitive insight.	[63,73,77]
Cognitive Remediation (CR)	Neurocognitive deficits.	Improved cognitive performance and functional outcomes.	[84,85,86]
Integrated Interventions	Self-disturbances, metacognition, and psychosocial functioning.	Greater improvements in recovery through combining cognitive and psychosocial approaches.	[87]

## Data Availability

No new data were created or analyzed in this study. Data sharing is not applicable to this article.

## Abbreviations

CR	Cognitive Remediation
DMN	Default Mode Network
EASE	Examination of Anomalous Self Experience
MAS-A	Metacognition Assessment Scale–Abbreviated
MCT	Metacognitive Training
MERIT	Metacognitive Reflection and Insight Therapy
SSDs	Schizophrenia Spectrum Disorders
WHO	World Health Organization
